# Steady-state global optimization of metabolic non-linear dynamic models through recasting into power-law canonical models

**DOI:** 10.1186/1752-0509-5-137

**Published:** 2011-08-25

**Authors:** Carlos Pozo, Alberto Marín-Sanguino, Rui Alves, Gonzalo Guillén-Gosálbez, Laureano Jiménez, Albert Sorribas

**Affiliations:** 1 Departament d'Enginyeria Química, Universitat Rovira i Virgili, Avinguda Països Catalans 26, 43007-Tarragona, Spain; 2 Technische Universität München. Fachgebiet für Systembiotechnologie, Boltzmannstr. 15 85748 Garching, Germany; 3 Departament de Ciències Mèdiques Bàsiques, Institut de Recerca Biomèdica de Lleida (IRBLLEIDA), Universitat de Lleida, Montserrat Roig 2, 25008 Lleida, Spain

## Abstract

**Background:**

Design of newly engineered microbial strains for biotechnological purposes would greatly benefit from the development of realistic mathematical models for the processes to be optimized. Such models can then be analyzed and, with the development and application of appropriate optimization techniques, one could identify the modifications that need to be made to the organism in order to achieve the desired biotechnological goal. As appropriate models to perform such an analysis are necessarily non-linear and typically non-convex, finding their global optimum is a challenging task. Canonical modeling techniques, such as Generalized Mass Action (GMA) models based on the power-law formalism, offer a possible solution to this problem because they have a mathematical structure that enables the development of specific algorithms for global optimization.

**Results:**

Based on the GMA canonical representation, we have developed in previous works a highly efficient optimization algorithm and a set of related strategies for understanding the evolution of adaptive responses in cellular metabolism. Here, we explore the possibility of recasting kinetic non-linear models into an equivalent GMA model, so that global optimization on the recast GMA model can be performed. With this technique, optimization is greatly facilitated and the results are transposable to the original non-linear problem. This procedure is straightforward for a particular class of non-linear models known as Saturable and Cooperative (SC) models that extend the power-law formalism to deal with saturation and cooperativity.

**Conclusions:**

Our results show that recasting non-linear kinetic models into GMA models is indeed an appropriate strategy that helps overcoming some of the numerical difficulties that arise during the global optimization task.

## 1 Background

Identifying optimization strategies for increasing strain productivity should be possible by applying optimization methods to detailed kinetic models of the target metabolism. Thus, a rational approach would pinpoint the changes to be done - e.g. by modulating gene expression - in order to achieve the desired biotechnological goals [1-4]. To build such models we can either start from a detailed description of the underlying processes (bottom-up strategy) or we can fit kinetic models to experimental data obtained *in vivo *(top-down strategy).

The bottom-up approach was the original strategy for model building in the biological sciences. Bottom-up kinetic models require information that is seldom available, despite the increasing amount of kinetic data contained in a growing set of databases (for example see [5,6] and the webpage http://kinetics.nist.gov/kinetics/index.jsp). Even in the best case scenarios where kinetic data are available, the data have often been obtained in different labs and under *in vitro *conditions that are hardly ever comparable or representative of the situation *in vivo*. In addition, models built using this strategy often fail to adequately reproduce the known behavior of the target system [7-10]. With the accumulation of time-series data, which were originally generated from the accurate measurement of transient responses, top-down modeling became viable as an alternative to the bottom-up strategy [11]. However, top-down modeling also faces important difficulties. For example, regulatory interactions between metabolites and enzymes are very poorly characterized and most metabolic maps lack such crucial information. Therefore, for a given network structure (i.e. a stoichiometric description) obtained from databases, a large number of alternative regulatory patterns may exist that account for the observed experimental data [12]. Model discrimination among the alternative regulatory patterns requires appropriate experimental design. However, this is seldom considered when performing the time series measurements. Last, but not the least, parameter identifiability in highly non-linear models can be problematic (for a review see [13]).

An additional issue that is common to models built using both strategies is that such detailed kinetic models include non-convexities that lead to the existence of multiple local optima in which standard non-linear optimization algorithms may get trapped during the search. Several stochastic and deterministic global optimization methods have been proposed to overcome this limitation [14]. Deterministic methods, which are the only ones that can rigorously guarantee global optimality, rely on the use of convex envelopes or underestimators to formulate lower-bounding convex problems that are typically combined with spatial branch and bound strategies. Most of these methods are general purpose and assume special structures in the continuous terms of the mathematical model. Because of this, they can encounter numerical difficulties in specific metabolic engineering systems that require the optimization of kinetic models with a large number of non-convexities of different nature.

Given all these issues, it is hardly surprising that linear stoichiometric models have emerged as the most popular tool to analyze genome-wide metabolic networks using optimization techniques. Linear optimization problems can be solved using very fast and efficient algorithms [15,16] that are implemented in almost every kind of computer, ranging from laptops to cloud computing centers. In addition, such models require a relatively small amount of information.

The possibility of condensing information about a very large network in a compact form enabled stoichiometric models to provide interesting insights in many different cases. However, the apparent simplicity in building and analyzing stoichiometric models comes at the cost of neglecting regulatory signals, metabolite levels and dynamic constraints. Accounting for these features in a dynamic way requires using more detailed, non-linear, mathematical models [17,18].

These models go a step further than stoichiometric models by incorporating regulatory influences through a set of ordinary differential equations that can account for the system's dynamics. Building such models is often impossible because the appropriate functional form that needs to be used to describe the dynamical behavior of specific processes is in general unknown. Modeling strategies based on systematic approximated kinetic representations, such as power-laws [19-22], Saturating and Cooperative [23], or convenience kinetics [24], side-step this issue by providing uniform forms that are guaranteed to be accurate over a range of conditions and reduce the amount of information required to build the models. Because of the regularity in the form of the mathematical function, models based on approximate formalisms can be automatically built from the reaction scheme of the target system. The model parameters can subsequently be estimated from experimental data using different procedures [13,25].

Although building and analyzing of comprehensive genome-wide detailed models is still not viable in most cases (see however [26,27]), developing ways to extend large scale optimization analysis to larger and more realistic non-linear kinetic models is an important part of the future of systems biology [18]. In fact, the optimization of certain types of non-linear problems can already be solved very efficiently and geometric programming problems with up to 1,000 variables and 10,000 constraints can be solved in minutes on a personal computer.

Efficient global optimization techniques are available for power-law models [1,28-30], either in S-system form or in Generalized Mass Action (GMA) form (for a review see [31]). In the case of S-system models, a simple logarithmic transformation brings the model to a linear form [1]. In the case of GMA models, the problem can be efficiently solved using branch-and-bound [28,32] and outer-approximation techniques [29,30].

The usefulness of the global optimization techniques developed for GMA models has been shown in the analysis of the adaptive response of yeast to heat shock [29,33]. In essence, starting with a GMA model and considering a set of constraints on flux and metabolite values, we can obtain: (i) The pattern of enzyme activities that maximizes a given objective, (ii) The region of feasible changes in enzyme activities so that the model fulfills a set of constraints on fluxes, metabolites, maximum allowable change in activity, etc., and (iii) A heat map of how the objective function changes within the feasible region. These results share some similarities with those produced with stoichiometric models, but incorporate many additional features.

Based on ideas similar to those that led to the development of the power-law formalism, Sorribas et al. [23] proposed a new Saturable and Cooperative (SC) formalism, that extends the power-law representation to include cooperativity and saturation. Although models built using this new formalism loses some of the simplicity inherent to the analysis of S-systems and GMA models, they tend to be accurate over a wider range of conditions than both the S-System and GMA representations [23]. Thus, it is important to enlarge the scope of global optimization methods developed for power-law models in order to deal with the SC formalism and analyze under which situations the later models behave better than the former.

Optimization of SC models faces a number of practical problems common to kinetic non-linear models [34,35]. To sidestep these problems, and in order to be able to use global optimization methods developed for power-law models, we will use a technique called recasting. Recasting permits the exact transformation of a continuous non-linear model with an arbitrary form into a canonical GMA model [36,37]. This transformation is typically performed by increasing the number of variables of the original model. Through this technique, arbitrary non-linear models can be represented using a canonical form such as GMA or S-system that can be used for simulation and optimization purposes, which opens the door for effectively extending the optimization and feasibility analysis originally devised for GMA models to other detailed kinetic models.

In this paper, and as a first step to define a framework for optimization of non-linear models with arbitrary form and extend FBA and related approaches to detailed kinetic models, we shall show the practical utility of recasting SC models into GMA models for optimization purposes. This technique is similar to the symbolic reformulation algorithm proposed by Smith and Pantelides [38]. Our method, however, focuses on obtaining a power-law representation that greatly facilitates global optimization, instead of continuing with the recasting until converting the model to a standard form containing linear constraints and a set of nonlinearities corresponding to bilinear product, linear fractional, simple exponentiation and univariate function terms. After recasting the model to the canonical form, we can apply any of the optimization strategies we have presented for GMA models [29,32] to obtain the global optimum of the original SC problem.

## 2 Results

### 2.1 Global optimization of non-linear models through recasting

**Figure 1:**
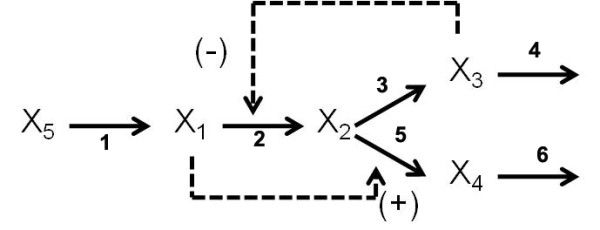
**Branched network with feedback and feedforward regulation.** X5 is a fixed external variable that can be varied at will. A GMA reference model is set-up by selecting appropriate parameters (see text).

For a proof of concept of the difficulties of global optimizing non-linear models and of the use of recasting for attaining practical solutions, we shall start by defining a reference biochemical network that corresponds to the reaction scheme in Figure 1. This hypothetical system has a source metabolite *X*_5 _and four internal metabolites. The network includes six reactions and a branch point. *X*_3 _acts as a feed-back inhibitor of the synthesis of *X*_2_, while *X*_1 _is an activator of the synthesis of *X*_4_.

The generic model for this system is:

(1) $$\begin{array}{lll} & {Ẋ}_{1}=v_{1}-v_{2}\; & \text{(1)}\\ & {Ẋ}_{2}=v_{2}-v_{3}-v_{5}\; & \text{(2)}\\ & {Ẋ}_{3}=v_{3}-v_{4}\; & \text{(3)}\\ & {Ẋ}_{4}=v_{5}-v_{6}\; & \text{(4)}\\ & \; & \text{(5)} \end{array}$$

Each of the velocities is a non-linear function of the involved metabolites. The SC representation, provides a systematic way for defining a functional model of this pathway. As a demonstrative example, let us suppose that the numerical model is:

(2) $$\begin{array}{lll} \frac{dX_{1}}{dt} & =\frac{20k_{1}X_{5}^{1}}{X_{5}^{1}+1}-\frac{40k_{2}X_{1}^{1}}{\left(X_{1}^{1}+1\right)X_{3}^{2}\left(1+\frac{1}{X_{3}^{2}}\right)}\; & \text{(1)}\\ \frac{dX_{2}}{dt} & =\frac{40k_{2}X_{1}^{1}}{\left(X_{1}^{1}+1\right)X_{3}^{2}\left(1+\frac{1}{X_{3}^{2}}\right)}-\frac{7.5k_{3}X_{2}^{2.5}}{X_{2}^{2.5}+0.25}\; & \text{(2)}\\ & \;-\frac{16k_{5}X_{1}^{1}X_{2}^{1}}{\left(X_{1}^{1}+1\right)\left(X_{2}^{1}+1\right)}\; & \text{(3)}\\ \frac{dX_{3}}{dt} & =\frac{7.5k_{3}X_{2}^{2.5}}{X_{2}^{2.5}+0.25}-\frac{12k_{4}X_{3}^{1}}{X_{3}^{1}+1}\; & \text{(4)}\\ \frac{dX_{4}}{dt} & =\frac{16k_{5}X_{1}^{1}X_{2}^{1}}{\left(X_{1}^{1}+1\right)\left(X_{2}^{1}+1\right)}-\frac{8k_{6}X_{4}^{1}}{X_{4}^{1}+1}\; & \text{(5)}\\ & \; & \text{(6)} \end{array}$$

In these equations, *k_r_*, *r *= 1,.., 6 is an auxiliary variable used to model changes in the enzyme activity. At the basal level, *k_r _*= 1 for all the reactions. During the optimization tasks, it is possible to limit the maximum change in gene expression by imposing a maximum allowable change in *k_r_*.

We shall now address the following questions:

(i) To what extent can general purpose global optimization methods be applied to SC models?, (ii) Given that a SC model can be recast as a GMA (rGMA), is this useful for optimization of the original SC model?, (iii) Are the results obtained with the rGMA equivalent to the results of the original SC model?, and (iv) What are the practical advantages of optimizing a rGMA model?.

### 2.2 Optimization goals

In order to address the questions posed at the end of the previous section we shall define the following optimizations tasks (note that changes in enzyme activities and metabolite concentrations are constrained between 0.2 ≤ *k_r _*≤ 5.0 and 0.1 ≤ *X_i _*≤ 10.0 respectively in all the instances unless otherwise specified):

• O1: What is the optimal pattern of changes in enzyme activities that maximizes the objective function in the new steady-state for a fixed value of *X*_5_?

• O2: What is the optimal pattern of changes in enzyme activities that maximizes the objective function in the new steady-state for a fixed value of *X*_5 _considering a maximum allowable variation of 10% in the steady-state values of the intermediaries?

• O3: What is the optimal pattern of changes in enzyme activities that maximizes the objective function in the new steady-state for a fixed value of *X*_5 _considering changes in the output flux from *X*_4 _of less than 10% with respect to its reference value?

• O4: What is the best set of changes, assuming that we can only manipulate three enzymes, that maximizes the objective function in the new steady-state for a fixed value of *X*_5 _considering a maximum variation of 10% in the steady-state values of the intermediaries?

Two different objective functions (OF), steady-state concentration of *X*_3 _and flux *v*_4_, have been considered for each optimization case, except for O3. This latter case has been optimized in terms only of the first objective (i.e., steady-state concentration of *X*_3_), because limits on *v*_4 _are already included in the formulation of the optimization problem.

### 2.3 Global optimization of SC models using BARON

We first address the optimization of the aforementioned model in their original SC form using state of the art global optimization techniques. The model was coded in the algebraic modeling system GAMS 23.0.2 and solved with the commercial global optimization package BARON v.8.1.5. on an Intel 1.2 GHz machine. An optimality gap (i.e., tolerance) of 0.2% was set in all the instances. As can be seen in Table 1, BARON produce results with an optimality gap (OG) below the specified tolerance.

**Table 1:** Results for the maximization of *X*_3 _and *v*_4 _and optimization goals O1-O4 using BARON v.8.1.5. for a tolerance of 0.2%.

O	*k*_1_	*k*_2_	*k*_3_	*k*_4_	*k*_5_	*k*_6_	*X*_3_	OG (%)	CPU (s)
1	0.26	5.00	4.97	0.20	0.20	0.54	8.30	0.20	136.17
2	0.20	0.24	0.22	0.20	0.21	0.20	1.10	0.00	0.06
3	0.60	5.00	5.00	0.53	0.20	0.27	5.39	0.20	96.39
4	0.99	1.15	1.00	0.96	1.00	1.00	1.10	0.00	1.42

**O**	***k*_1_**	***k*_2_**	***k*_3_**	***k*_4_**	***k*_5_**	***k*_6_**	***v*_4_**	**OG (%)**	**CPU (s)**

1	4.61	5.00	5.00	5.00	0.72	1.20	37.40	0.20	157.83
2	3.22	3.73	5.00	4.99	0.21	0.22	31.33	0.00	1.67
3	0.88	0.94	0.88	0.96	0.23	3.00	6.60	0.00	10.53
4	1.16	1.00	1.34	1.34	1.00	1.00	7.61	0.00	3.61

**Figure 2:**
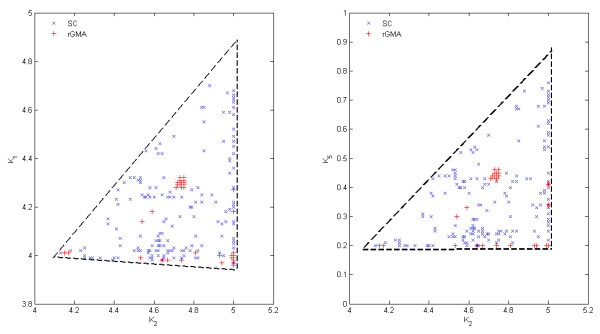
**Equivalent optimal solutions for the case S1-O1-v4.** Blue points indicates results on the original SC model obtained with BARON. Red points identify solutions obtained for the corresponding rGMA and OA method (see text for details).

Table 1 only shows one solution for each particular instance. However, BARON identified in each case a set of equivalent optima (i.e, solutions with the same objective function value) involving different changes in enzyme activities, which indicates that the optimization problem is somehow degenerated. This redundancy is a consequence of the system's structure and has practical implications. As an example, we have calculated some of these equivalent points for case O1-*v*_4 _using the *NumSol *option of BARON (see Figure 2). In particular, a well defined triangular region containing the changes in *k*_2 _and *k*_5_, and *k*_1 _and *k*_2 _that lead to the same objective function value is identified. Within these regions, one can decide which combination of changes should be selected based on additional cost arguments, as they all show the same performance in terms of the predefined objective function. This region could be further reduced by imposing additional constraints to the optimization.

### 2.4 Recasting SC models into GMA models

Any SC model can be recast into a GMA canonical model by introducing the auxiliary variables $z_{rj}=K_{rj}+X_{j}^{n_{rj}}$. Substitution and differentiation generates the following recast GMA (rGMA) model:

(3a) $$\begin{array}{c} \begin{array}{c} {Ẋ}_{i}=\overset{p}{\underset{r=1}{\sum }}\mu_{ir}V_{r}\overset{n+m}{\underset{j=1}{\prod }}X_{j}^{n_{rj}}z_{rj}^{-1} \end{array}\;\begin{array}{c} i=1,..,n \end{array}\\ \;\;\;\;\;\;\;\;\; \end{array}$$

(3b) $$\begin{array}{c} {ż}_{rj}=n_{rj}X_{j}^{n_{rj}-1}{Ẋ}_{j} \end{array}\;\begin{array}{c} r=1,..,p\\ j=1,...,n+m \end{array}$$

with appropriate initial conditions $X_{j}\left(0\right)=X_{j_{0}}$ and $z_{rj}\left(0\right)=K_{rj}+X_{j_{0}}^{n_{rj}}$.

For simulation purposes, model (3) is equivalent to the original SC model. As discussed in [36], a model recast into a GMA model has the same steady-state that the original non-linear model. The steady-state equations of the rGMA model can be expressed as:

(4a) $$\begin{array}{c} \overset{p}{\underset{r=1}{\sum }}\mu_{ir}V_{r}\overset{n+m}{\underset{j=1}{\prod }}X_{j}^{n_{rj}}z_{rj}^{-1}=0 \end{array}\;\begin{array}{c} i=1,..,n \end{array}$$

(4b) $$\begin{array}{c} n_{rj}X_{j}^{n_{rj}-1}{Ẋ}_{j}=0 \end{array}\;\begin{array}{c} r=1,..,p\\ j=1,...,n+m \end{array}$$

### 2.5 Steady-state optimization of SC models through recasting

The steady-state solutions of Eqn. (4b) satisfy also Eqn. (4a). Thus, for optimization purposes, the steady-state constraints of interest are:

(5a) $$\begin{array}{c} \overset{p}{\underset{r=1}{\sum }}\mu_{ir}V_{r}\overset{n+m}{\underset{j=1}{\prod }}X_{j}^{n_{rj}}z_{rj}^{-1}=0 \end{array}\;\begin{array}{c} i=1,..,n \end{array}$$

(5b) $$\begin{array}{c} K_{rj}+X_{j_{0}}^{n_{rj}}=z_{rj_{0}} \end{array}\;\begin{array}{c} r=1,..,p\\ j=1,...,n+m \end{array}$$

According to these results, the optimization problem can be stated as:

(6) $$\begin{array}{cc} min-OF & OF=\left\{X_{i}\text{ or }v_{r}\right\}\\ \text{s}\text{.t}\text{.} & \\ \sum_{r=1}^{p}\mu_{ir}k_{r}v_{r}\prod_{J=1}^{n+m}X_{j}^{n_{rj}}z_{rj}^{-1}=0 & i=1,..,n\\ & j=1,...,n+m\\ K_{rj}+X_{j_{0}}^{n_{rj}}=z_{rj_{0}} & r=1,..,p\\ & j=1,...,n+m\\ X_{iL}\leq X_{i}\leq X_{iU} & i=1,...,n\\ k_{rL}\leq k_{r}\leq k_{rU} & r=1,...,p\\ .....\;\text{additional}\;\text{constraints}\;........ & \end{array}$$

In our reference model, we shall consider the following constraints:

(7) $$\begin{array}{cc} min-OF & OF=\left\{X_{3},v_{4}\right\}\\ \text{s}\text{.t}\text{.} & \\ \sum_{r=1}^{p}\mu_{ir}k_{r}v_{r}\prod_{J=1}^{n+m}X_{j}^{n_{rj}}z_{rj}^{-1}=0 & i=1,..,n\\ K_{rj}+X_{j_{0}}^{n_{rj}}=z_{rj_{0}} & r=1,..,p\\ & j=1,...,n+m\\ \text{Specific}\;\text{constraints}\;\text{for}\;\text{each}\;\text{optimization}\;\text{task} & \\ \text{(O}1\text{,}\;\text{O}3\;\text{only)} & \\ 0.1\leq X_{i}\leq 10 & i=1,...,n\\ \begin{array}{c} \text{(O}1\text{,}\;\text{O}2\text{,}\;\text{O}3\;\text{only)} \end{array} & \\ 0.2\leq k_{r}\leq 5 & r=1,...,p\\ \begin{array}{c} \text{(O}2,\;\text{O}4\;\text{only)} \end{array} & \\ 0.9X_{i}^{BAS}\leq X_{i}\leq 1.1X_{i}^{BAS} & i=1,...,n\\ \begin{array}{c} \text{(O}3\;\text{only)}\;\text{and}\;\text{(}OF:X_{3}\;\text{only)} \end{array} & \\ 0.9v_{4}^{BAS}\leq v_{4}\leq 1.1v_{4}^{BAS} & \\ \begin{array}{c} \text{(O}4\;\text{only)} \end{array} & \\ k_{r}=k_{r1}+k_{r2}+k_{r3} & r=1,...,p\\ k_{r}^{LB}y_{r1}\leq k_{r1}\leq \left(1-\delta \right)y_{r1} & r=1,...,p\\ \left(1-\delta \right)y_{r2}\leq k_{r2}\leq \left(1+\delta \right)y_{r2} & r=1,...,p\\ \left(1+\delta \right)y_{r3}\leq k_{r3}\leq k_{r}^{UB}y_{r3} & r=1,...,p\\ y_{r1}+y_{r2}+y_{r3}=1 & r=1,...,p\\ \sum_{r=1}^{p}y_{r1}+\sum_{r=1}^{p}y_{r3}\leq ME=3 & \end{array}$$

Once the problem has been recast into a rGMA, its mathematical structure can be exploited in order to improve the efficiency of the solution procedure, as demonstrated by the authors in previous works. This problem has a GMA form except for the auxiliary constraint 5b, which is required to recast the SC into the rGMA. This constraint can be easily handled by means of relaxation techniques and exponential transformations similar to those used by the authors in their global optimization algorithms for pure GMA models [32,33]. In particular, two algorithms were developed for the global optimization of GMA models: a customized outer-approximation (OA, [30]) and a tailored spatial branch-and-bound (sBB, [32]). The authors showed that the numerical performance of these methods depends on the specific problem being solved, and that none of them is clearly better than the other one. Here, we use the OA algorithm to solve 6, as this method proved to be faster than sBB for problems of smaller size ([32]). Again, the main body of the algorithm was coded in GAMS 23.0.2, using CPLEX 11.2.1 as MILP solver for the master subproblems and CONOPT 3.14 s as NLP solver for the slave subproblems of the algorithm. For a fair comparison, we also set a tolerance of 0.2%, the same as when using BARON.

As can be seen in Table 2, the optimization of the rGMA formulation using our customized OA yields similar results to those obtained when BARON is applied to the original SC model. In some cases, significant reductions in computational time are attained with our OA algorithm. While BARON took a total time of 407.68 CPU seconds for solving the 8 instances, the customized OA algorithm solved the same problems in 8.5 CPU seconds.

**Table 2:** Results for the maximization of *X*_3 _and *v*_4 _using the rGMA model and optimization goals O1-O4 using the customized OA for a tolerance of 0.2%.

O	*k*_1_	*k*_2_	*k*_3_	*k*_4_	*k*_5_	*k*_6_	*X*_3_	OG (%)	CPU (s)
1	0.26	5.00	5.00	0.20	0.20	0.20	8.30	0.20	2.94
2	0.21	0.22	0.21	0.20	0.20	0.20	1.10	0.00	0.06
3	0.60	5.00	5.00	0.53	0.20	0.24	5.40	0.13	2.35
4	1.00	1.05	0.97	0.92	1.00	1.00	1.10	0.00	0.23

**O**	***k*_1_**	***k*_2_**	***k*_3_**	***k*_4_**	***k*_5_**	***k*_6_**	***v*_4_**	**OG (%)**	**CPU (s)**

1	3.96	5.00	5.00	5.00	0.20	2.99	37.47	0.00	0.16
2	3.22	3.55	5.00	4.99	0.20	0.21	31.33	0.17	0.66
3	0.68	1.79	1.12	1.27	0.20	0.21	6.60	0.00	0.12
4	1.16	1.00	1.34	1.34	1.00	1.00	7.61	0.11	1.98

Note that the objective function values obtained with the SC and rGMA models only differ within the tolerance imposed. In some cases, discrepancies regarding the enzymatic profiles calculated are observed mainly due to the system's structure, that is, to the fact that the problem contains multiple solutions attaining the same performance in terms of objective function value but involving different enzymatic configurations, as discussed in section 2.3.

To further investigate this issue, we apply the multi-solution capability of BARON to the rGMA model (Figure 2). Again, different equivalent optima are obtained, but this time the dispersion of the equivalent solutions generated for a given case tend to concentrate either in the center or in the extremes of the region containing the solutions with the same objective function value calculated with the SC model.

The region illustrated in Figure 2 should not be misunderstood as a feasibility region. In fact, solutions do exist outside this region, but they lead to worse objective function values. To further clarify this issue, we consider a grid of values for *k*_2 _and *k*_5 _in the region defined by constraints 4 ≤ *k*_2 _≤ 5 and 0.2 ≤ *k*_5 _≤ 0.8, and solve the optimization problem within each cell applying BARON to the SC model, and our OA to the rGMA model. Recall that these linear constraints define a region that contains that in Figure 2. The results obtained in this optimization are illustrated in Tables 3 and 4, and are exactly equal for both methods. However, the CPU time is much lower when using our OA algorithm applied to rGMA (11,811 CPU seconds for generating all the points with BARON applied to the SC model vs 17 CPU seconds with the customized OA applied to the rGMA model; as shown in Tables 5 and 6).

**Table 3:** Results (objective function) of the optimization of case O1- *v*_4 _for specific regions of *k*_2 _and *k*_5 _obtained with BARON for the SC model.

*k*_5_/*k*_2_	1	2	3	4	5	6	7	8
8	36.50	36.71	36.90	37.08	37.24	37.37	37.47	37.47
7	36.62	36.83	37.02	37.19	37.34	37.46	37.47	37.47
6	36.75	36.95	37.14	37.31	37.44	37.47	37.47	37.47
5	36.88	37.08	37.26	37.41	37.47	37.47	37.47	37.47
4	37.02	37.21	37.38	37.47	37.47	37.47	37.47	37.47
3	37.15	37.34	37.47	37.47	37.47	37.47	37.47	37.47
2	37.29	37.46	37.47	37.47	37.47	37.47	37.47	37.47
1	37.43	37.47	37.47	37.47	37.47	37.47	37.47	37.47

Domain of each *k_r_*(4 ≤ *k*_2 _≤ 5;0.2 ≤ *k*_5 _≤ 0.8) has been split into 8 intervals with equal width.

**Table 4:** Results (objective function) of the optimization of case O1-*v*_4 _for specific regions of *k*_2 _and *k*_5 _obtained with the customized OA for the rGMA model.

*k*_5_--*k*_2_	1	2	3	4	5	6	7	8
8	36.50	36.71	36.90	37.08	37.24	37.37	37.47	37.47
7	36.62	36.83	37.02	37.19	37.34	37.46	37.47	37.47
6	36.75	36.95	37.14	37.31	37.44	37.47	37.47	37.47
5	36.88	37.08	37.26	37.41	37.47	37.47	37.47	37.47
4	37.02	37.21	37.38	37.47	37.47	37.47	37.47	37.47
3	37.15	37.34	37.47	37.47	37.47	37.47	37.47	37.47
2	37.29	37.46	37.47	37.47	37.47	37.47	37.47	37.47
1	37.43	37.47	37.47	37.47	37.47	37.47	37.47	37.47

Domain of each *k*_r_(4 ≤ *k*_2 _≤ 5;0.2 ≤ *k*_5 _≤ 0.8) has been split into 8 intervals with equal width.

**Table 5:** Results (CPU time in seconds) of the optimization of case O1- *v*_4 _for specific regions of *k*_2 _and *k*_5 _obtained with BARON for the SC model.

*k*_5_/*k*_2_	1	2	3	4	5	6	7	8
8	212.53	308.53	185.64	201.80	222.30	201.53	139.16	178.31
7	194.81	161.16	215.80	196.81	344.73	243.02	0.03	174.81
6	234.30	203.75	147.08	180.69	328.34	254.42	304.11	280.53
5	212.08	282.41	329.33	237.34	208.02	292.27	200.00	154.62
4	288.00	160.14	92.94	235.80	172.69	147.14	56.11	150.28
3	125.56	111.17	150.27	187.52	337.97	158.16	112.66	264.12
2	239.70	190.59	100.03	138.47	106.38	205.14	119.39	246.34
1	140.42	102.12	80.45	21.69	73.12	96.61	89.94	80.03

Domain of each *k*_r_(4 ≤ *k*_2 _≤ 5;0.2 ≤ *k*_5 _≤ 0.8) has been split into 8 intervals with equal width.

**Table 6:** Results (CPU time in seconds) of the optimization of case O1-*v*_4 _for specific regions of *k*_2 _and *k*_5 _obtained with the customized OA for the rGMA model.

*k*_5_/*k*_2_	1	2	3	4	5	6	7	8
8	0.13	0.27	0.23	0.18	0.17	0.19	0.28	0.28
7	0.26	0.28	0.28	0.26	0.28	0.23	0.32	0.25
6	0.32	0.30	0.28	0.28	0.27	0.23	0.19	0.25
5	0.31	0.21	0.25	0.25	0.26	0.28	0.27	0.29
4	0.25	0.27	0.32	0.30	0.25	0.27	0.26	0.28
3	0.20	0.22	0.28	0.28	0.29	0.30	0.19	0.53
2	0.28	0.25	0.19	0.19	0.22	0.17	0.30	0.25
1	0.23	0.24	0.26	0.27	0.23	0.21	0.24	0.31

Domain of each *k*_r_(4 ≤ *k*_2 _≤ 5;0.2 ≤ *k*_5 _≤ 0.8) has been split into 8 intervals with equal width.

### 2.6 Difficult optimization tasks can be solved via recasting

The reference model can be optimized either by general purpose techniques or by rGMA specific methods such as the customized OA. However, even with this simple example, we may encounter instances that are hard to solve using standard techniques. Consider, for instance, the same reaction scheme as before but this time with the alternative parameters indicated in the following model:

(8) $$\begin{array}{lll} & \frac{dX_{1}}{dt}=\frac{11.11k_{1}X_{5}^{2.86}}{X_{5}^{2.86}+0.81}\; & \text{(1)}\\ & \;\;-\frac{12.35k_{2}X_{1}^{1.54}}{\left(X_{1}^{1.54}+0.61\right)X_{3}^{6.81}\left(0.11+\frac{1}{X_{3}^{6.81}}\right)}\; & \text{(2)}\\ & \frac{dX_{2}}{dt}=\frac{12.35k_{2}X_{1}^{1.54}}{\left(X_{1}^{1.54}+0.61\right)X_{3}^{6.81}\left(0.11+\frac{1}{X_{3}^{6.81}}\right)}\; & \text{(3)}\\ & \;\;-\frac{4.44k_{3}X_{2}^{4.14}}{X_{2}^{4.14}+0.11}\; & \text{(4)}\\ & \;\;-\frac{7.41k_{5}X_{1}^{0.51}X_{2}^{26.51}}{\left(X_{1}^{0.51}+0.19\right)\left(X_{2}^{26.51}+0.11\right)}\; & \text{(5)}\\ & \frac{dX_{3}}{dt}=\frac{4.44k_{3}X_{2}^{4.14}}{X_{2}^{4.14}+0.11}-\frac{4.44k_{4}X_{3}^{4.14}}{X_{3}^{4.14}+0.11}\; & \text{(6)}\\ & \frac{dX_{4}}{dt}=\frac{7.41k_{5}X_{1}^{0.51}X_{2}^{26.51}}{\left(X_{1}^{0.51}+0.19\right)\left(X_{2}^{26.51}+0.11\right)}\; & \text{(7)}\\ & \;\;-\frac{6.67k_{6}X_{4}^{1.57}}{X_{4}^{1.57}+1.40}\; & \text{(8)}\\ & \; & \text{(9)} \end{array}$$

The optimization task of interest being:

• O5: Which is the optimal pattern of changes in enzyme activities that maximize *v*_6 _in the new steady-state for a fixed value of *X*_5 _and considering the following constraints?

(9) $$\begin{array}{cc} 0.3\leq X_{1}\leq 30 & \\ 0.1\leq X_{2}\leq 10 & \\ 0.1\leq X_{3}\leq 10 & \\ 0.6\leq X_{4}\leq 50 & \\ 0.1\leq k_{r}\leq 20 & r=1,...,p \end{array}$$

When BARON is employed to solve this case using the native SC form, it cannot reduce the optimality gap below the specified tolerance after 1 hour of CPU time. In contrast, when the model is recast into its rGMA form and our OA method is applied, the global optimum can be determined with an optimality gap of 2% in 10.95 seconds (see Table 7). This illustrates both, the utility of using the rGMA as a canonical form for dealing with the optimization of SC models, and the computational efficiency of our global optimization methods specifically designed to take advantage of the mathematical structure of the GMA.

**Table 7:** Results of the optimization of model 8 with BARON (SC model) and the customized OA (rGMA model).

Solver	*k*_1_	*k*_2_	*k*_3_	*k*_4_	*k*_5_	*k*_6_	OF	OG (%)	CPU (s)
BARON (SC)	6.24	5.16	0.46	0.6	8.46	9.09	60.36	45.18	3600
OA (rGMA)	6.25	5.17	0.45	0.6	8.44	9.1	60.46	2.18	10.95

## 3 Discussion

While experimental tools to manipulate gene expression are already available, there is no established set of guidelines on how these tools can be used to achieve a certain goal. So far, two main difficulties have prevented model driven optimization from becoming a standard in providing such guidelines: (i) the lack of information to build detailed kinetic models and (ii) the computational difficulties that arise upon the optimization of such models. The latter can be exemplified by the application of mixed integer non-linear optimization techniques (MINLP) in the context of kinetic models presented in [34,35]. In such cases, the optimization task showed to be computationally very demanding and global optimality could not be guaranteed in many cases. We propose that using models with a standardized structure may offer a solution to both problems. On one hand, approximate kinetics, such as the SC formalism, can provide very accurate approximations and retain key features of the system like saturation and cooperativity. On the other hand, these formalisms can be automatically recast into GMA form and using efficient global optimization methods developed specifically for this canonical representation. Although this technique will certainly have limitations, our previous results indicate that it can be applied to models of moderate complexity without major problems [32]. Optimization of GMA models comprising up to 60 reactions and 40 metabolites offer no limitation to our technique. We have shown how these methods can be easily used to optimize SC via recasting into rGMA models while still being quite efficient.

Our results can be of particular interest for dealing with multicriteria optimization on realistic models. This kind of problems are relevant when exploring the adaptive response to changing conditions, were conflictive goals may be at play [39,40]. Particularly, we should notice that several multi-objective optimization techniques, such as the weighted sum or epsilon constraint methods [41] are based on solving a set of auxiliary single-objective problems. These approaches could directly benefit from the numerical advances presented in this work. This kind of problems are relevant when exploring the adaptive response to changing conditions, were conflictive goals may be on play [39,40]. The highly efficient OA algorithm applied to rGMA models provide a practical way for extending multicriteria optimization methods, for instance as used in [39], to non-linear kinetic models. It is in principle possible to make use of methods such as ours to analyze the optimality of large scale dynamic systems much in the same way that Flux Balance Analysis can be applied to analyze the stoichiometry of an organism on a genomic scale. To make this possible, however, extensive experimental and modeling efforts would be required to characterize the most important properties of the involved processes. In fact, we anticipate that practical limitations to apply the techniques presented here in solving larger problems will be dominated by the lack of information about the component processes and metabolites rather than by the technical capacity of the optimization technique presented here. Although a complete kinetic characterization of the processes in a complete metabolic network may yet be far, information on kinetic orders and saturation fractions is easier to obtain. In this context, the SC formalism provides a sound approximation that results in a mathematical representation useful for simulation and optimization through recasting.

## 4 Conclusions

We expect that the possibility of building models using non-linear approximate formalisms and of subsequently optimizing these models will trigger interest in the experimental characterization of the components of cellular metabolism. After the genomic explosion, we need to step back and begin to measure enzyme activities, metabolite levels, and regulatory signals on a larger scale than we used to do before, if we want to understand the emergence of the dynamic properties of biological systems and to be able to develop successful biotechnological applications.

## 5 Methods

### 5.1 Modelling strategies

The process of model building and optimization can be used to understand how a system should be changed in order to achieve specific biotechnological goals or how the same system has evolved in order to more efficiently execute a given biological function. Different trade-offs are considered during the modeling process. On the one hand, one wants to use models that are as simple as possible to guarantee numerical tractability. Unfortunately simplifications may lead to models whose accuracy is only ensured for a limited range of physiological conditions. On the other hand, models that are very detailed and accurate over a wide range of physiological conditions are typically more difficult to analyze and optimize. Needless to say, the type of modeling strategy and the model one chooses to implement have a large impact on the results of the analysis. The most widely used strategies in the context of optimization are: (1) Stoichiometric models, (2) Kinetic models, and (3) Approximated models.

The three strategies have as a starting point a set of ordinary differential equations, in which the dependent variables or nodes are the chemical species whose dynamical behavior one is interested in studying. For a system with *n *dependent variables, *p *processes and *m *independent variables, the node equations are written as follows:

(10) $${Ẋ}_{i}=\overset{p}{\underset{r=1}{\sum }}\mu_{ir}v_{r}\;\;i=1,..,n$$

*μ_ir _*stands for the stoichiometry of each metabolite *X_i _*in each reaction *r *with respect to metabolite *i *and can be derived from the reaction scheme.

At this stage, the various strategies begin to differ in the way that they implement and analyze the equations. Typically, Flux balance analysis (FBA) and related techniques consider only the steady state behavior of the system, and treat *v_r _*as a variable whose value can be changed in order to optimize specific steady state constraints. To accomplish this, FBA-like methods attempt to find solutions for the following system of linear equations:

(11) $$0=\overset{p}{\underset{r=1}{\sum }}\mu_{ir}v_{r}\;i=1,..,n$$

This system of equations is solved under different assumptions. A typical problem is that of understanding the effect of knocking out different genes from the system. This analysis can be performed by setting *v_r _*= 0 for the process(es) that depend on the product of the genes that are knocked out. Once these constraints are set, linear optimization techniques can be used to identify the region of the variable space that satisfies the steady state and optimizes at the same time a set of specific measurable aspects of the systems [42-44]. It must be noted that FBA analysis of Eqn. (11) does not account for the regulatory effects that can result from gene knockout and it cannot be used to predict changes in metabolic concentrations and temporal responses. Thus, optimization constraints are limited to steady-state fluxes [15].

To overcome these limitations, we must use more complex kinetic models where the effect of changing the values of the variables on the fluxes is taken into account. This requires defining a functional form for each *v_r _*in Eqn. (10). Often, this functional form is drawn from a number of classical enzyme kinetic rate-laws. As a result, we use an approximate expression for the kinetic behavior of each elementary process whose form depends on the underlying mechanism of the process. The reason for this is that the classical rate laws are rational functions of the variables and they are built upon different types of simplifying assumptions on the detailed mechanism of the reactions. Such assumptions range from considering that the elementary chemical steps of the catalytic process occur at very different timescales to assuming that the concentration of the catalyst and of the reactants differ in orders of magnitude. Thus, rate laws such as the popular Michaelis-Menten are approximations to the actual mechanism in specific conditions. However, more often than not, one does not have enough information to judge if such conditions meet those one is trying to model. Thus, using rational enzyme kinetics in models lacks a sound theoretical ground. In fact, within the complex architecture of the intracellular milieu, many of the assumptions that justify these classical rate-laws may not hold [45-47]. Even in the best case scenario where a detailed kinetic model using classical enzyme kinetics can be derived and numerically identified, it may be hard to globally optimize that model using general purpose algorithms. As we will show here, available optimization techniques may fail to solve fairly trivial optimization tasks even in simple models. These numerical difficulties can be overcome by defining reformulated models based on canonical representations that are easier to handle using customized global optimization algorithms devised for specific canonical functional forms.

As an alternative, theoretically well supported canonical representations can be derived using approximation theory. One type of such representations are power-law models. In a power-law model, each *v_r _*in Eqn. (10) is approximated as [19,21]:

(12) $$\begin{array}{lll} & v_{r}\left(X_{1},..,X_{n},...X_{n+m}\right)\; & \text{(1)}\\ & \;\;\;\;\;\;\approx \gamma_{r}\overset{n+m}{\underset{j=1}{\prod }}X_{j}^{f_{jr}}r=1,..,p\; & \text{(2)}\\ & \; & \text{(3)} \end{array}$$

This approximation is derived at a given operating point $\left(X_{1_{0}},X_{2_{0}},..,X_{{\left(n+m\right)}_{0}}\right)$ as a first-order Taylor series representation of the target function in log-log space. This approximation can generate models with different representations. The two that are most commonly used are the S-system representation and the GMA representation. The S-system representation is obtained by lumping the various processes that contribute to the synthesis of a given metabolite into a global process of synthesis $V_{i}^{+}$ and those that contribute to the utilization of a given metabolite into a global degradation process $V_{i}^{-}$:

(13) $$\begin{array}{lll} & {Ẋ}_{i}=\overset{p}{\underset{r=1}{\sum }}\mu_{ir}v_{r}\; & \text{(1)}\\ & \;=\overset{p}{\underset{r=1}{\sum }}\mu_{ir}^{+}v_{r}-\overset{p}{\underset{r=1}{\sum }}\mu_{ir}^{-}v_{r}\; & \text{(2)}\\ & \;=V_{i}^{+}-V_{i}^{-}\;\;\;\;i=1,..,n\; & \text{(3)}\\ & \; & \text{(4)} \end{array}$$

Then, the aggregated processes are represented by power-law functions:

(14) $$\begin{array}{c} {Ẋ}_{i}=\alpha_{i}\overset{n+m}{\underset{j=1}{\prod }}X_{j}^{g_{ij}}-\\ \;\;\beta_{i}\overset{n+m}{\underset{j=1}{\prod }}X_{j}^{h_{ij}}\;i=1,..,n \end{array}$$

Alternatively, the GMA form is obtained representing each individual *v_r _*as a power-law:

(15) $$\begin{array}{lll} & {Ẋ}_{i}=\overset{p}{\underset{r=1}{\sum }}\mu_{ir}v_{r}\; & \text{(1)}\\ & =\overset{p}{\underset{r=1}{\sum }}\left(\mu_{ir}\gamma_{r}\overset{n+m}{\underset{j=1}{\prod }}X_{j}^{f_{rj}}\right)\;i=1,..,n\; & \text{(2)}\\ & \; & \text{(3)} \end{array}$$

The parameters in these representations have a clear physical interpretation. Kinetic orders, the exponents in the power-laws, are local sensitivities of the fluxes, either individual (*f_rj _*for *v_r_*) or aggregated (*g_ij _*for $V_{i}^{+}$ and *h_ij _*for $V_{i}^{-}$), with respect to *X_j_*. Rate-constants (*α_i_*, *β_i _*and *γ_r_*) are parameters that are computed so that the flux in the model at steady state is equal to the operating flux at the operating point for the metabolites. Parameter estimation techniques have been developed so that power-law parameters can be calculated from experimental measurements [13]. It should also be noted that the use of estimation procedures (i.e., least-squares), alternate regression or similar procedures to estimate power-law parameters from dynamic curves lead to a power-law representation that is no longer local according to the classical definition [48-50]. Those models may, by definition, slightly improve their accuracy over strictly local models.

To complement the power-law approach, the Saturable and Cooperative (SC) formalism was introduced by Sorribas et al. [23] as an extension of the ideas that led to the power-law formalism. The SC representation of a given velocity is:

(16) $$v_{r}\left(X_{1},..,X_{n},...X_{n+m}\right)\approx \frac{V_{r}\overset{n+m}{\underset{j=1}{\prod }}X_{j}^{n_{rj}}}{\overset{n+m}{\underset{j=1}{\prod }}\left(K_{rj}+X_{j}^{n_{rj}}\right)}$$

This representation can be obtained from a power-law model defined at a given operating point *X*_0 _= (*X*_10_,.., *X*_(*n *+ *m*)0_) through the following relationships:

(17) $$\begin{array}{cc} n_{rj}=\frac{f_{rj}}{1-p_{rj}} & \;r=1,..,p\\ & \;j=1,..,n+m \end{array}$$

(18) $$\begin{array}{cc} K_{rj}=\frac{1-p_{rj}}{p_{rj}}X_{i0}^{n_{rj}} & \;r=1,..,p\\ & \;j=1,..,n+m \end{array}$$

Thus SC uses the same information as the power-law except for the new parameters *p_rj _*(saturation fractions), which are defined as:

(19) $$\begin{array}{cc} p_{rj}=v_{r0}∕V_{rj} & \;r=1,..,p\\ & \;j=1,..,n+m \end{array}$$

where *v_r0 _*= *v_r_*(*X*_10_,.., *X_n0_*,... *X*_(*n *+ *m*)0_) and *V_rj _*is either the limit velocity (saturation) when *X_j _*→ ∞ if *n_rj _*> 0, or the limit velocity when *X_j _*→ 0 if *n_rj _*< 0.

Using SC models for global optimization can raise some numerical issues. These difficulties can be avoided to a large extent by recasting SC models into a canonical GMA model, through the introduction of auxiliary variables, as will be shown in the next section.

### 5.2 Recasting non-linear models into power-law canonical models by increasing the number of variables

Non-linear models can be *exactly *recast into GMA or S-system models through the use of auxiliary variables [36]. As a result, the final model is an exact representation of the original model, written in a canonical form. In other words, the resulting GMA model is not an approximation to the original model: it is an exact replica of it. To avoid confusion, hereafter, we refer to a GMA model that exactly recasts another as an rGMA model.

**Figure 3:**
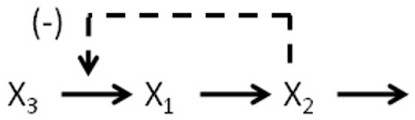
A simple linear network.

As a very simple introductory example, consider a linear pathway with two internal metabolites *X*_1 _and *X*_2 _and a source metabolite *X*_3 _(Figure 3). In this pathway, *X*_2 _is a competitive inhibitor of the synthesis of *X*_1 _from the source metabolite. A generic model using Michaelis-Menten kinetic functions, assuming a competitive inhibition of the first reaction by *X*_2_, can be written as:

(20) $$\begin{array}{lll} & {Ẋ}_{1}=\frac{V_{1}X_{3}}{K_{1}\left(1+K_{i}∕X_{2}\right)+X_{3}}\; & \text{(1)}\\ & \;-\frac{V_{2}X_{1}}{K_{2}+X_{1}}\; & \text{(2)}\\ & {Ẋ}_{2}=\frac{V_{2}X_{1}}{K_{2}+X_{1}}-\frac{V_{3}X_{2}^{2}}{K_{3}^{2}+X_{2}^{2}}\; & \text{(3)}\\ & \; & \text{(4)} \end{array}$$

in which *X*_3 _is an externally fixed variable.

Recasting this model as a rGMA can be done as follows. First, let us define three new variables:

(21) $$\begin{array}{lll} & X_{4}=K_{1}\left(1+Ki∕X_{2}\right)+X_{3}\; & \text{(1)}\\ & X_{5}=K_{2}+X_{1}\; & \text{(2)}\\ & X_{6}=K_{3}^{2}+X_{2}^{2}\; & \text{(3)}\\ & \; & \text{(4)} \end{array}$$

We can now write the model in 20 as:

(22) $$\begin{array}{lll} {Ẋ}_{1}=V_{1}X_{3}X_{4}^{-1}-V_{2}X_{1}X_{5}^{-1} & \; & \text{(1)}\\ {Ẋ}_{2}=V_{2}X_{1}X_{5}^{-1}-V_{3}X_{2}^{2}X_{6}^{-1} & \; & \text{(2)}\\ & \; & \text{(3)} \end{array}$$

with initial conditions $X_{1}\left(0\right)=X_{1_{0}}$ and $X_{2}\left(0\right)=X_{2_{0}}$.

To complete the recasting we must now provide the equations that follow the change in the new variables over time. These are given by the following equations:

(23) $$\begin{array}{ccc} {Ẋ}_{4} & =-\frac{K_{1}K_{i}{Ẋ}_{2}}{X_{2}^{2}} & \\ & =V_{3}K_{1}K_{i}X_{6}^{-1}-V_{2}K_{1}K_{i}X_{1}X_{5}^{-1}\\ X_{2}^{-2}{Ẋ}_{5} & ={Ẋ}_{1}\\ & =V_{1}X_{3}X_{4}^{-1}-V_{2}X_{1}X_{5}^{-1}\\ {Ẋ}_{6} & =2X_{2}{Ẋ}_{2}\\ & =2V_{2}X_{1}X_{2}X_{5}^{-1}-2V_{3}X_{2}^{3}X_{6}^{-1} \end{array}$$

with initial conditions $X_{4}\left(0\right)=K_{1}\left(1+Ki\text{/}X_{2_{0}}\right)+X_{3_{0}}$, $X_{5}\left(0\right)=K_{2}+X_{1_{0}}$, and $X_{6}\left(0\right)=K_{3}^{2}+X_{2_{0}}^{2}$.

The resulting rGMA model (22-23) is an exact representation of model in (20). Hence, for a set of appropriate initial conditions, the simulation of the dynamic response using either the model recast as a rGMA or the original model will produce the same trajectory. In principle, any non-linear model can be recast into a rGMA following a similar procedure [36]. This can be extremely useful, because it allows for the application of tailored global optimization procedures originally devised for GMA models [28-30,32,51,52] to generic non-linear models.

## Competing interests

The authors declare that they have no competing interests.

## Authors' contributions

AM-S suggested the potential utility of recasting for optimizing non-linear kinetic models. AS and AM-S elaborate on the recasting of SC models and planned the work. CP, GG-G and LJ implemented the OA algorithm and worked out the technical solution for applying it to a rGMA model. CP and GG-G performed the optimization tasks. AS and RA defined the reference model and obtained the numerical parameters used in the paper. All authors read and approved the final manuscript.
